# Protocol for a Pilot Two-Arm Crossover Randomized Controlled Trial of the ACTIVE Intervention for Older Adults with and Without Mild Dementia and Their Care Partners

**DOI:** 10.3390/jcm15041341

**Published:** 2026-02-08

**Authors:** Oluwaseun Adeyemi, Tracy Chippendale, Joshua Chodosh, Dowin Boatright

**Affiliations:** 1 Department of Emergency Medicine, New York University Grossman School of Medicine, New York, NY 10016, USA; 2 Department of Occupational Therapy, New York University Steinhardt School of Culture, Education, and Human Development, New York, NY 10003, USA; 3 Department of Population Health, New York University Grossman School of Medicine, New York, NY 10016, USA; 4 Department of Medicine, New York University Grossman School of Medicine, New York, NY 10016, USA; 5 Veterans Affairs New York Harbor Healthcare System, New York, NY 10010, USA

**Keywords:** ACTIVE, older adults, Alzheimer’s disease and Alzheimer’s disease-related dementias, pilot, quality of life, randomized control trial, feasibility, efficacy, care recipient–care partner dyad

## Abstract

**Background:** Older adults, including those with Alzheimer’s disease and Alzheimer’s disease-related dementias (AD/ADRD), face barriers in maintaining regular physical activity, which increases their fall risk and reduces their quality of life. The Activity Tracking, Care Partner Co-Participation, Text Reminders, Instructional Education, Video-Guided Physical Rehabilitation, and Exercise trial aims to improve physical activity among older adults. This pilot study aims to assess the feasibility and preliminary efficacy of the ACTIVE intervention. **Methods:** ACTIVE is a multi-method, two-arm randomized, analyst-blinded crossover pilot trial with an embedded qualitative component. We will recruit 50 community-dwelling dyads (older adult–care partner, n = 100), with and without mild AD/ADRD, in a 1:1 ratio. Each dyad will be randomized to the intervention or control arm, stratified by AD/ADRD diagnosis. The intervention arm will receive activity tracking, motivational texts, walking exercises, educational videos, and video-guided physical rehabilitation sessions, while the control arm will receive only activity tracking. The intervention will run in two halves: a three-week intervention/control, a one-week crossover phase, and a three-week control/intervention phase. The quantitative outcome measures will include feasibility measures (recruitment, adoption, adherence, acceptability, fidelity, and retention), and measures of preliminary efficacy (activity metrics, fall risk and fear of falling, functional limitations, and quality of life). Qualitatively, we will assess participants’ experiences, and facilitators and barriers to engagement in physical activity through semi-structured dyadic interviews and thematic analysis. **Conclusions:** This pilot study will generate data on the feasibility and preliminary efficacy of the ACTIVE trial. Findings will inform a full-scale implementation trial.

## 1. Introduction

Physical activity is a well-established determinant of health and functional independence among older adults [1,2]. However, older adults face significant barriers to maintaining regular physical activity, including symptoms of chronic disease, mobility limitations, and challenges with motivation, activity initiation, and maintenance [3,4,5]. These challenges are particularly pronounced among persons living with Alzheimer’s disease or Alzheimer’s disease-related dementias (AD/ADRD), who are at a 2- to 4-fold higher risk of falls and poorer quality of life compared to those without AD/ADRD [6,7]. Malnutrition, a geriatric syndrome associated with sarcopenia, functional vulnerability, and biological frailty [8], further contributes to reduced functional status and quality of life in older populations [9]. Despite evidence supporting the benefits of physical activity in reducing fall risk [10,11], and enhancing overall quality of life [12,13], adherence remains a challenge, particularly for older adults with AD/ADRD who may require additional support to maintain activity routines.

Improving physical activity among older adults requires approaches grounded in established behavioral theory. The Social Cognitive Theory (SCT) is one such theory, and it describes behavior as the product of continuous interactions among personal, behavioral, and environmental factors [14]. Within the personal domain, constructs such as self-efficacy and outcome expectations shape an individual’s confidence and beliefs about the benefits of being active [15,16]. The behavioral domain includes self-regulation skills, such as goal-setting, self-monitoring, and adjusting one’s actions based on feedback, that support sustained engagement in physical activity [15,16]. The environmental domain encompasses external influences, such as social support, modeling, and reinforcement, that can facilitate adherence to physical activity [15,16].

**Figure 1:**
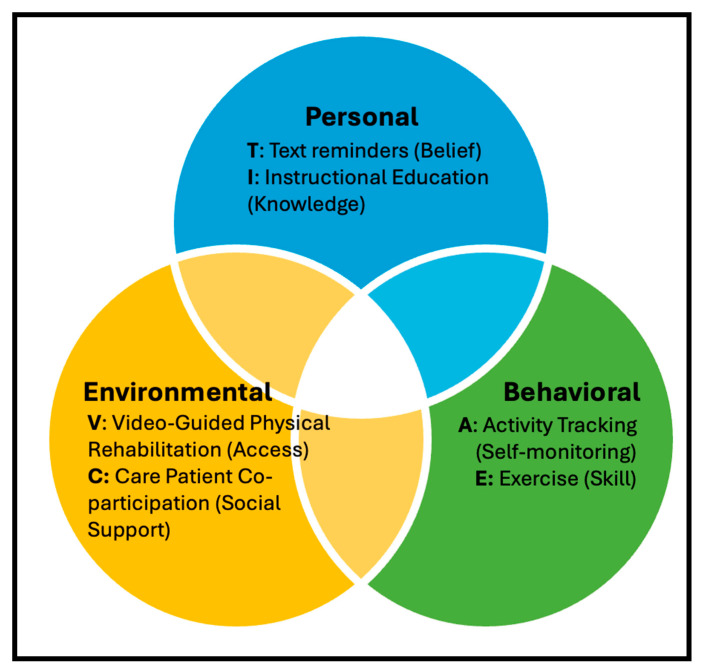
Visual display of the Social Cognitive Theory used in designing the Activity tracking, Care partner co-participation, Text reminders, Instructional education, Video-Guided Physical Rehabilitation, and Exercise (ACTIVE) Intervention.

Using the SCT, we designed a multi-component intervention that comprises Activity tracking, Care partner co-participation, Text reminders, Instructional education, Video-Guided Physical Rehabilitation, and Exercise (ACTIVE). ACTIVE targets the personal domain through instructional education (I) and text reminders (T), the behavioral domain through structured walking exercise (E), and activity tracking (A), and the environmental domain through care partner co-participation (C) and video-guided physical rehabilitation (V) (Figure 1). These components are particularly relevant for older adults with mild AD/ADRD, whose cognitive impairment may not substantially impact their understanding of activity instructions.

Empirical evidence suggests that wearable activity trackers, such as Fitbit smartwatches, improve self-monitoring, with preliminary studies showing higher step counts and greater adherence to physical activity among older adults [17,18,19]. Motivational text messages have been shown to reinforce behavior, provide reminders, and sustain activity among older adults [20,21,22]. Virtual physical therapy offers a scalable approach to improving strength, balance, and mobility by delivering structured, remotely tailored exercise programs [23,24,25]. Walking exercise provides a simple, evidence-based strategy to increase daily physical activity, improve cardiovascular fitness, reduce the severity of fall injuries, and reduce the fear of falling [21,26]. Care partner involvement further enhances adherence, provides social support, and ensures safety, with dyadic interventions demonstrating improved outcomes in physical activity and health management [27,28]. Together, these components of the ACTIVE intervention target both behavioral and functional pathways to reduce fall risk and improve quality of life among older adults.

By integrating wearable technology, video-guided physical rehabilitation, walking exercise, text reminders, education, and care partner co-participation, the ACTIVE intervention has the potential to improve physical activity engagement, reduce fall risk, and improve the quality of life of older adults with and without mild AD/ADRD. The project will contribute to the growing evidence on digital health interventions for aging populations and inform the development of scalable strategies to promote physical activity among older adults (care recipients) and their care partners. To our knowledge, no prior intervention has employed a theory-guided, multi-dimensional approach to simultaneously improve physical activity, reduce fall risk, and improve quality of life for older care recipient–care partner dyads with and without mild AD/ADRD. This pilot study has two aims. First, we aim to examine the feasibility (i.e., recruitment, adoption, adherence, acceptability, fidelity, and retention) of the ACTIVE intervention among older care recipient–care partner dyads with and without mild AD/ADRD. Second, we aim to evaluate the preliminary efficacy of the intervention on perception, attitudes, and engagement in physical activity, fall risk and fear of falling, and quality of life in these dyads.

## 2. Materials and Methods

For this remote, home-based, technology-assisted intervention, we will conduct a multi-methods pilot study using a two-arm, crossover, analyst-blinded randomized controlled trial with embedded qualitative interviews. The study population will consist of community-dwelling older care recipients and their care partners (dyads) with and without mild AD/ADRD. Our study population will be recruited from ResearchMatch, a national, NIH-funded volunteer registry that connects researchers with individuals interested in participating in health-related studies [29]. It has over 120,000 volunteers, including over 13,000 older adults and 700 with self-reported AD/ADRD [29]. We will send recruitment emails weekly and enroll participants on a rolling basis until we reach our target sample size.

### 2.1. Eligibility Criteria

Eligible care recipients will be aged 65 years or older, English or Spanish-speaking, community-dwelling, and have no contraindications to engaging in physical activity. They must be willing to wear a smartwatch, receive text messages, watch intervention-related videos, and participate in video-guided physical rehabilitation sessions and walking exercises. Care recipients will be ineligible if they do not have a care partner or have severe cardiac, neurologic, or musculoskeletal conditions (including, but not limited to, a recent joint replacement or recent or untreated fractures in any part of the body) that limit participation in walking exercise or smartwatch use. Also, they will be excluded if they have moderate to severe dementia and are unable to operate or manage a smartwatch even with care partner support. Additionally, they will be excluded if they have no internet, phone, or any devices capable of receiving text messages or watching educational or physical therapy-related videos.

Similarly, eligible care partners must be at least 18 years old. They must be the legally authorized representative of the care recipient when the care recipient lacks the capacity to consent. They must live with or within five miles of the care recipient and be able to co-participate in all aspects of the ACTIVE intervention. They will be ineligible if they have severe cardiac, neurologic, or musculoskeletal conditions (including but not limited to recent or untreated fractures in any part of the body), limiting participation in walking exercise or smartwatch use. Also, they will be excluded if they have moderate to severe dementia, are unable to operate or manage a Fitbit smartwatch, have no internet, phone, or any device capable of receiving text messages or watching educational or physical therapy-related videos.

### 2.2. Screening, Recruitment, and Enrollment

Screening, recruitment, and enrollment will be rolling for 12 months or until we obtain 50 dyads—25 older adults and care partners without mild dementia (25) and with mild dementia (25). Screening will start with a recruitment email that includes the eligibility requirements sent to potential participants registered on ResearchMatch. Those interested in the study will provide their email addresses, and a formal email with a screening and recruitment survey link will be sent to them. The screening survey will include a brief questionnaire requesting their age, caregiving status, language preference, willingness to participate in a physical activity intervention and/or survey, and access to a smartphone or computer. Those who meet the eligibility criteria will gain access to the recruitment survey.

The recruitment survey will request their email and phone number, ask for the name, email address, and phone number of their care partner/care recipient, and request a preferred date for the video enrollment session. Text messages and emails will be automatically sent to the listed care recipient and care partner to confirm participation in the enrollment video session. Within 48 h of the care recipient and the care partner completing the survey, the dyad will receive emails and text messages confirming the date and time of the video enrollment session.

This video enrollment session will include a member of the study team and the two members of the dyad. If either member is unavailable, the study team member will reschedule the call. Both members must be visible in the video call to establish a dyadic enrollment. During the video enrollment session, the study team member will explain the study intervention in lay terms and obtain consent for participation. For care recipients with self-reported dementia, the study team member will administer the Ascertain Dementia-8 questionnaire (AD-8; score ≤ 4) [30] or the Clinical Dementia Rating (CDR; score ≤ 1) [31] to verify whether dementia is mild. For those with an AD-8 score of ≤4 or a CDR ≤ 1, the study team member will assess capacity to consent using the University of California San Diego Brief Assessment of Capacity to Consent (UBACC) [32]. A score of 14.5 out of 20 is required to be eligible to consent [32]. Those with a score ≤ 14.5 will require a care partner who is their legally authorized representative to provide consent for participation.

Dyads that consent will complete a self-administered baseline survey (discussed below). Also, during the video call, the study team will guide the care recipient and care partner in creating user accounts on myACTIVEsteps.com and Fitabase, and on the Fitbit smartwatch (discussed below), using their preferred names, user identities (IDs), and phone numbers. Each video enrollment session is expected to last between 45 and 60 min. After the video call, the study materials (Fitbit smartwatch, educational materials on fall prevention, and instructions for accessing the online videos and using the smartwatch) will be sent to study participants within 1 week of enrollment. All surveys and consents will be administered remotely on Research Electronic Data Capture (REDCap) [33]. All study participants will be informed that de-identified ACTIVE study data may be used for future ancillary studies without additional participant contact, subject to IRB approval.

### 2.3. Randomization

Participants will be assigned to study arms after completion of the baseline enrollment survey. The study staff who enrolls each participant will notify the study biostatistician, who will provide the allocation. Randomization will be conducted using a computer-generated, permuted block randomization sequence stratified by dyad type (with or without mild AD/ADRD). Within each stratum, randomization will be performed using permuted blocks of 4 to ensure balanced group sizes. Both members of the dyad will be randomized together to the same intervention or control arm. The randomization sequence will be generated in advance by the study biostatistician using a random number generator and securely maintained within the study database, accessible only after baseline data collection is completed. After the third week of intervention, we will have a one-week crossover period, and all participants will switch to the alternate study arm. This one-week crossover period is a washout period to minimize the effects of promptings and motivations produced by the text messages, rather than to eliminate retained knowledge or behavior change. Group assignments will remain linked to each participant’s unique study ID throughout the study. The analyst will remain blinded to group assignments until data cleaning, feasibility, and preliminary efficacy analyses are complete.

### 2.4. Study Materials

While all surveys will be delivered using REDCap, we will use an additional six primary tools to deliver the ACTIVE intervention and obtain study data. These tools include (1.) Fitbit/Fitabase, (2.) myACTIVEsteps.com, (3.) printed educational materials, (4.) instructional education videos, (5.) physical therapy videos, and (6.) the Zoom video conferencing application.

Fitbit Inspire 3 Smartwatch and Fitabase: Participants will receive a pre-configured de-identified Fitbit Inspire 3 smartwatch to passively collect activity data, including daily step counts, activity intensity (daily duration of light, moderate, and intense physical activities), and daily heart rate variability. Fitabase, a secure HIPAA-compliant data management platform, will link each device to a study-specific dashboard and capture all transmitted Fitbit data in real time. Also, motivational text messages will be sent from the Fitabase platform to all study participants.

myACTIVEsteps.com Platform: This secure website houses all asynchronous video-guided physical rehabilitation sessions and instructional educational modules [34]. The website automatically captures engagement metrics, including logins, video access, and watch time (in minutes) for both the video-guided physical rehabilitation sessions and educational modules.

Printed Educational Materials: Care recipients and partners will receive printed copies of the Centers for Disease Control and Prevention’s fall prevention resources—“Stay Independent” [35], and “Family Caregivers: Protect Your Loved Ones from Falling” [36].

Instructional Educational Videos: Care recipients and care partners will access three-minute educational videos during the intervention period. The content of these videos was developed by the study team and is grounded in evidence-based fall prevention strategies from the Centers for Disease Control and Prevention (CDC) STEADI initiative [35,36,37]. Care recipient videos will focus on (1) benefits of moving, (2) safe home exercises, (3) fall prevention, (4) pain management, (5) building routines, and (6) long-term strength and independence. Care partners will receive parallel educational modules emphasizing (1) their role in supervision, (2) setting up safe environments, (3) recognizing risks, (4) encouraging safe activity, (5) reinforcing progress, and (6) transitioning support as independence improves.

Video-Guided Physical Rehabilitation videos: Care recipients and their care partners will have access to nine asynchronous evidence-based video-guided physical rehabilitation videos created by the National Institute on Aging (US), the Falls Prevention and Healthy Aging Network (Australia), and the National Health Service Inform Falls Information Zone (UK). Video content covers gait, strength, and balance training, and each video lasts 10 to 15 min. The study team obtained permission to use these publicly available videos.

Zoom Videoconferencing: Qualitative interviews will be conducted via Zoom, using audio-only recording. Recordings will be transcribed using a secure AI-assisted transcription service, and transcripts will be de-identified before analysis.

Study materials and surveys are available in both English and Spanish. Bilingual staff will conduct interviews and provide support for Spanish-speaking dyads. Expert physical therapy educational videos are delivered in English, and Spanish subtitles will be available for Spanish-speaking participants. All participant-facing materials, including surveys, consents, videos, and platform content translated from English to Spanish, will be validated by bilingual experts prior to use.

### 2.5. Intervention Design

The ACTIVE intervention consists of six co-occurring events as follows (Table 1):

Activity Tracking: Care recipients and their care partners will be provided with a wrist-worn Fitbit smartwatch to wear during waking hours for at least 8 h per day. Activity metrics will be tracked for care recipients and care partners on Fitabase.

Care partner co-participation: Care partners will play active, structured roles in the intervention. These roles include (1.) Safety oversight, (2.) Co-engagement, (3.) Education access and (4.) Motivation and supervision. Care partners will be required to ensure their care recipients use the smartwatch consistently, walk safely, and adhere to exercise protocols (safety oversight). They will participate in walking exercises and video-guided physical rehabilitation sessions together with their care recipient (co-participation). They will view educational videos to understand how best to support care recipients to stay active in safe environments (education). Lastly, they will encourage adherence, monitor fatigue, and help adjust routines as independence improves (motivation and supervision). Care partner engagement will be assessed using a multimodal approach, including platform-based analytics (login frequency, module completion), structured prompts embedded within myACTIVEsteps, and brief scheduled daily text message check-ins to capture real-time participation and support activities.

Text reminders: Validated motivational text messages will be delivered to both care recipients and care partners at scheduled morning times tailored to each dyad. These messages were developed using Self-Determination Theory. They encompass nine activity-based categories: (1) exceeding the daily activity goal; (2) meeting the daily target; (3) slightly below target; (4) low activity; (5) no activity data received; (6) improvement in activity compared with the prior day; (7) decrease in activity compared with the prior day; (8) three or more consecutive days of high activity; and (9) three or more consecutive days of low activity. Details of the development and validation of the motivated text messages have been published [38]. Message delivery will be dynamically tailored based on each participant’s recorded activity from the preceding day (e.g., exceeded goal, low activity, no data, or sustained inactivity). Messages will be brief, supportive, and action-oriented, serving as daily prompts to reinforce engagement and sustained physical activity.

Instructional education: Care recipients and care partners will be assigned six brief instructional videos (2–3 min each), organized into twice-weekly educational modules delivered over the three-week intervention period. Video engagement will be automatically tracked through the myACTIVEsteps platform. A video will be considered completed if at least 70% of the total viewing time is reached.

Virtual physical therapy: Care recipients and their care partners will have access to nine evidence-based video-guided physical rehabilitation modules over three weeks—three videos per week, each lasting 10–15 min. Duration of engagement and percent completion will be tracked remotely on the website. A video session will be considered completed if at least 70% of the total viewing time is reached. We expect both care partners and their care recipients to participate in each session. At the start of each session, the user ID of the person logging in will be required, and the person will attest that the care partner/care recipient is available during the session.

Exercise (Walking Program): The walking program emphasizes daily walking, ideally outdoors or in safe indoor settings (hallways, local community centers, schools, malls, walking paths). Care recipients will be encouraged to walk with their care partner at least once daily, starting with 10–15 min and gradually increasing to 30 min per day, 5 days a week. Care partners will monitor pacing, ensure environmental safety, and help reinforce adherence. Walking activity will be tracked by the smartwatch and reinforced through motivational texts.

### 2.6. Intervention and Control Groups

Dyad participants randomized to the intervention arm will experience all six components of the ACTIVE intervention—Activity tracking, Care partner co-participation, Text reminders, Instructional education, Video-Guided Physical Rehabilitation, and Exercise. Dyad participants randomized to the control group will receive the Fitbit smartwatch only for physical activity tracking. They will be instructed to wear the smartwatch daily during waking hours. They will not receive motivational text messages, instructional education, or video-guided physical rehabilitation content during the intervention period. Dyad participants in the intervention and control arms may choose to discontinue participation at their own request without penalty. If either member of a dyad chooses to discontinue participation, the entire dyad will be withdrawn from the study. Participants will also be encouraged to continue their regular healthcare and daily routines.

### 2.7. Study Procedure

**Figure 2:**
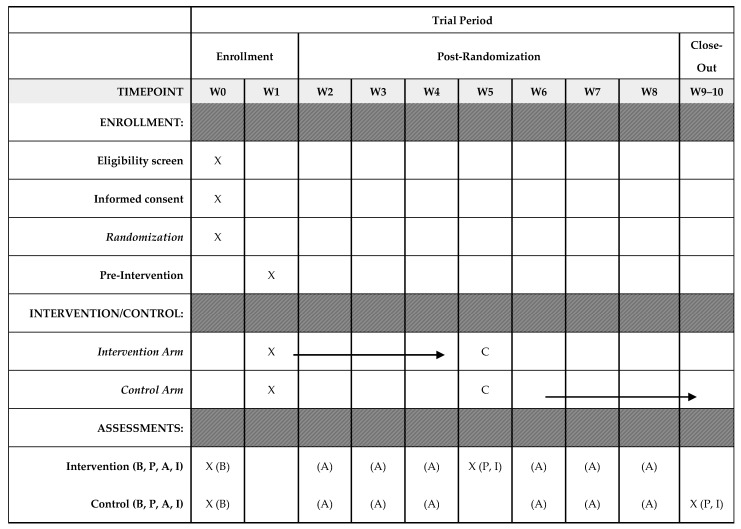
Timeline and schedule of enrollment, interventions, and assessments. A: Activity Tracking; B: Baseline Survey; C: Crossover Period; P: Post-Intervention Survey; I: Interviews; W: Week; X: Point where event occurs.

The study will run for 10 weeks (Figure 2), starting with a one-week pre-intervention phase, followed by three weeks of intervention (or control), one week crossover phase, three weeks of control (or intervention), and a two-week post-intervention phase. The 3-week intervention period was selected to balance feasibility assessment with participant burden, consistent with pilot study methodology [39,40]. This duration provides adequate time to evaluate key feasibility outcomes while generating preliminary effect estimates to inform sample-size calculations for a definitive trial. Prior digital health intervention pilots have used a similar intervention period to assess the feasibility of technology-based interventions [41,42,43].

Week 1 (Pre-Intervention): The study team will call the dyad to confirm receipt of the study materials and ensure: (1) the appropriate wearing of the Fitbit smartwatches and data capture, (2) receipt of motivational text messages, and (3) access to the educational videos (Table 2). Data collected during this week will not be used for the study.

Weeks 2–4 (Phase I Intervention/Control): The intervention will start with dyads randomized to the intervention arms. Remote tracking of physical activity data will continue in the control arm.

Week 5 (Crossover/Washout): Participants will switch arms (intervention to control and vice versa). Activity data during this crossover week will not be analyzed (Table 2). Participants in the intervention arm will complete six post-intervention surveys (discussed below), and a few will participate in interviews.

Weeks 6–8 (Phase II Intervention/Control): Participants will continue in their new assignments (opposite of initial condition).

Weeks 9–10 (Post-Intervention): Crossover participants in the intervention arm will undergo post-intervention surveys and interviews. All participants will have access to the educational videos and video-guided physical rehabilitation sessions upon completion of the study (Table 2).

### 2.8. Sample Size Determination

For this pilot study, the trial is not powered to detect clinical efficacy but to provide adequate precision for estimating feasibility metrics and to inform the design of a future fully powered effectiveness–implementation trial [44]. We will enroll 50 dyads (older adult + care partner), a recommended sample size for behavioral pilot trials and digitally delivered rehabilitation interventions [44,45,46]. A sample of 50 dyads (n = 100) provides adequate precision to estimate key feasibility parameters that guide go/no-go decisions.

For a feasibility outcome, the unadjusted standard error (SE), assuming independence, is estimated using the formula in Equation (1). Using an expected proportion of 0.75 (e.g., completion of ≥75% of virtual PT modules), the unadjusted SE is 0.043.

(1) SE=p (1−p)n

To account for dyadic clustering, we assume an intra-cluster correlation (ICC) of 0.1–0.3, which is typical for partner dyads [47,48]. Using the design effect formula in Equation (2), an ICC of 0.2, and a cluster size (m) of 2, the design effect is 1.2, and the cluster-adjusted SE (Equation (3)) is 0.047. The cluster-adjusted 95% CI for a 75% completion rate (Equation (4)) is therefore 0.66–0.84.

Design Effect = 1 + (m − 1) × ICC(2)

Cluster-adjusted Standard Error (adjusted SE) = Unadjusted SE × √(Design Effect)(3)

(4) 95% CI=p±1.96×adjusted SE

This level of precision is appropriate for feasibility-focused pilot research, where the goal is to estimate plausible ranges and plan thresholds for an adequately powered future trial.

### 2.9. Data Collection, Management, and Retention

Across the study period, we will collect (1) feasibility measures, (2) preliminary efficacy measures, (3) covariates, and (4) qualitative interview data from older adults and their care partners (Table 3). Data will be captured through surveys, Fitbit/Fitabase activity monitoring, myACTIVEsteps.com engagement metrics, and Zoom-based interviews.

All data will be coded with unique study IDs and stored securely on access-restricted institutional servers, with access limited to authorized study personnel. No direct personal identifiers will be collected, and only de-identified data will be shared for analysis or future research. Data integrity will be maintained through standardized collection protocols, regular quality checks, and audit trails. Study data will be retained in accordance with institutional and federal requirements.

Participant retention will be promoted through proactive monitoring, reminders, and flexible follow-up scheduling. Even in cases of partial discontinuation, key outcomes will be collected when possible, including remote data capture for surveys and activity tracking.

### 2.10. Outcome Measures

Consistent with the study objectives, this pilot study includes two broad categories of outcomes: Feasibility Measures and Preliminary Efficacy Measures. The outcome variables, measurement metrics, and assessment time points are detailed below

Feasibility Outcome Measures:

The feasibility of the ACTIVE intervention will be evaluated in accordance with NIH guidelines for pilot studies [40], with a focus on recruitment, adoption, adherence, acceptability, fidelity, and retention (Table 3). Feasibility outcomes will be assessed separately by AD/ADRD status and by intervention and control arms.

Recruitment will be defined as the proportion of dyads recruited and randomized to the study, with a target of at least 75%. Adoption will be defined as the initial engagement in week one for smartwatch use, motivational text interaction, video-guided physical rehabilitation session initiation, and viewing educational videos, with a target of at least 75%. Adherence will be defined as sustained engagement during the three-week intervention period, with targets of at least 75% for days of smartwatch use, number of completed video-guided physical rehabilitation sessions, and number of educational videos viewed.

Acceptability will be assessed at weeks 5 and 9 using the System Usability Scale and the revised Technology Acceptance Model. The System Usability Scale is a 10-item survey (Cronbach alpha (α) = 0.90), and higher scores indicate higher usability of the specific study tool [49,50]. The revised Technology Acceptance Model is a 10-item scale (α = 0.89), and higher scores indicate greater acceptance of the specific technology [51,52]. The System Usability Scale and the revised Technology Acceptance Model surveys will be used to assess participants’ acceptability of (1.) the website (myACTIVEsteps.com), (2.) the Fitbit smartwatch, (3.) motivational texts, (4.) educational videos, and (5.) video-guided physical rehabilitation. Acceptability will be defined as a System Usability Scale score of at least 68 and a revised Technology Acceptance Model score of at least 4.0. [49,50,51,52]. Fidelity will measure the proportion of motivational and educational text notifications successfully delivered, with a target of at least 90%. Retention will be defined as the proportion of dyads that complete the study, with a target of at least 75%.

Preliminary Efficacy Outcome Measures:

Preliminary efficacy will focus on changes in activity metrics and psychosocial measures. The activity metrics will include daily step counts, daily metabolic equivalent of task, and daily average heart rate variability. These measures are pre-computed metrics on Fitabase. The psychosocial measures will include perceptions, attitudes, and practices related to physical activity, fall risk, fear of falling, functional limitations, and quality of life.

We will assess perceptions, attitudes, and practices regarding physical activity using the Perception of Physical Activity Benefits for Older Adults scale (α = 0.92), the Attitude towards Physical Activity Scale (α = 0.77), and the Physical Activity Practice Scale (α = 0.91) [53]. These perception, attitude, and practice scales each comprise 14, seven, and nine items, respectively. Higher scores on the scales indicate higher perceived benefits, a more positive attitude, and more engagement in physical activities [53].

We will define fall risk using the Stopping Elderly Accidents, Deaths, and Injuries (STEADI)—a 12-item scale (Area Under Curve: 0.91) [37,54]. Higher STEADI scores indicate higher fall risk [37,54]. Fear of falling will be measured using the 16-item Falls Efficacy Scale–International (FES-I; α = 0.96) [55]. Higher FES-I scores indicate greater fear of falling [55]. Functional limitations will be measured using the 6-item Katz Activities of Daily Living (α = 0.85) and the 8-item Instrumental Activities of Daily Living surveys (α = 0.94) [56,57]. Higher scores on these activity surveys indicate greater independence in basic and instrumental activities of daily living [56,57]. Quality of life will be assessed using the Physical and Mental Quality of Life Short Form-12 Health Survey (α = 0.87–0.90) [58]. Higher scores on the Short Form-12 indicate better physical and mental quality of life [58].

### 2.11. Covariates

Covariates will include sociodemographic and health survey data collected from the older adult and care partner at enrollment. This sociodemographic and health survey includes age, sex, race/ethnicity, education, insurance status, marital status, living situation, the Clinical Frailty Scale score [59,60], and the Charlson Comorbidity Index [61,62]. AD/ADRD status (mild AD/ADRD vs. no AD/ADRD) will be used as a stratification variable.

### 2.12. Data Collection by Study Phase

At baseline, dyads will complete sociodemographic and health surveys and background efficacy measures (Figure 2). During the mid-intervention assessment (intervention arm only), care recipients and care partners will repeat all baseline surveys (excluding the sociodemographic and health surveys) and additionally complete the revised Technology Acceptance Model and the System Usability Scale. At the crossover phase, selected dyads who began in the intervention arm will participate in qualitative interviews. At the end-of-study assessment, dyads who completed the delayed intervention will repeat all surveys, and some will be selected for qualitative interviews.

### 2.13. Analysis Plan

Handling of Missing Data: We anticipate low levels of missing data, which will be assessed for randomness using Little’s test [63,64]. Multiple imputation with chained equations will be used when appropriate [65,66,67], with sensitivity analyses to evaluate robustness of results. For feasibility outcomes, missing data will be minimized through proactive monitoring and participant reminders, and patterns will be summarized descriptively. For preliminary efficacy analyses, linear mixed-effects models will accommodate incomplete longitudinal data under an intention-to-treat framework. The percent and potential impact of missing data will be reported.

Feasibility Assessment: Six feasibility outcomes (recruitment, adoption, adherence, acceptability, fidelity, retention): will be summarized overall and stratified by AD/ADRD status (dyads with mild AD/ADRD and dyads without mild AD/ADRD) and by participant role within the dyad (care recipient vs. care partner). We will report the proportions and mean distributions, including 95% confidence intervals, for the feasibility measures, and descriptively compare these values with the preset benchmark (Table 3). A “go” decision will be made if the majority of benchmarks are met, a “conditional go” if some but not all benchmarks are achieved (with justification for modifications), and a “stop” decision if most benchmarks are not met. Feasibility outcomes will be assessed separately by AD/ADRD status and by intervention and control arms. Differences in feasibility outcomes by AD/ADRD status and by participant role will be explored descriptively using mean differences and differences in proportions with 95% confidence intervals.

Preliminary Efficacy Assessment: Using linear mixed-effects models, we will estimate the effect size of the ACTIVE intervention’s preliminary efficacy on the three activity metrics and seven post-intervention behavioral measures (listed above). The effect size will be estimated from the pre-crossover phase (Equation (5)). Full six-week mixed-effects models are used to evaluate whether longitudinal patterns remain consistent with the pre-crossover estimate, after accounting for the impact of the crossover and the sequence of intervention (Equation (6)). The random intercept will account for both within-dyad correlation and repeated measures. Fixed effects will include treatment (intervention vs. control), sequence (intervention first or last), baseline values of the outcomes, dementia status (mild AD/ADRD vs. no AD/ADRD), treatment × dementia interaction, period (pre-crossover vs. post-crossover), treatment × period interaction, and covariates to assess potential differential effects. For participant *i* in dyad *d* at time *t* (t = baseline, follow-ups), the outcome will follow the mixed effect model in Equations (1) and (2), as follows:

(5) $${\;Y}_{idt}=\;\beta_{0}+\;\beta_{1}{Trt}_{i}+\;\beta_{3}{Time}_{t}+\;\beta_{5}Y_{id0}+\;\beta_{6}{Dem}_{i}+\;\beta_{7}\left({Trt}_{i}\;\times \;{Dem}_{i}\right)+\;\tau^{T}X_{i}+\;b_{d}+\;b_{i\left(d\right)}+\;\in_{idt}\;\;$$

(6) $$Y_{idt}=\beta_{0}+\beta_{1}{Trt}_{i}+\beta_{2}{Seq}_{i}+\beta_{3}{Time}_{t}+\beta_{4}{Period}_{t}+\beta_{5}Y_{id0}+\beta_{6}{Dem}_{i}+\beta_{7}\left({Trt}_{i}\times {Dem}_{i}\right)+\beta_{8}\left({Trt}_{i}\times {Period}_{i}\right)+\tau^{T}X_{i}+b_{d}+b_{i(d)}+\in_{id\;\;\;\;\;\;\;\;}$$

where *Y_idt_* = outcome, *β*_0_ = fixed intercept, *Trt_i_* = treatment indicator, *Seq_i_* = sequence indicator, *Y_id_*_0_ = baseline value of the outcome, *Demi* = dementia status, *Trt × Dem* = treatment-by-dementia interaction, *Trt × Period =* treatment-by-crossover interaction, γ^⊤^*X_i_* = coefficient of covariates, and *b_d_* and *b_i(d)_* are the random intercept for dyad and individual nested within dyad, respectively. Treatment condition (intervention vs. control) and sequence (intervention-first vs. intervention-second) will be included as fixed effects to estimate the average intervention effect and to account for between-participant differences related to assignment order, respectively. Time will be modeled as a continuous weekly measure to capture trends over the six-week follow-up. Period will be modeled separately as a categorical indicator of pre- versus post-crossover phase to distinguish underlying activity trajectories from changes associated with withdrawal of active intervention components. Given the anticipated persistence of learning effects due to the short one-week washout period, the treatment × period interaction will be used to characterize maintenance or attenuation of intervention effects rather than to assume their absence. All analytical models will follow the intention-to-treat principle, and effect sizes with 95% confidence intervals will be reported to inform power calculations for a future definitive trial.

Qualitative Assessment: Semi-structured interviews will be conducted with dyads after completing the intervention phases. Dyads initially randomized to the intervention arm will be interviewed at crossover, while those starting in the control arm will be interviewed after completing the delayed intervention. Interviews will explore barriers and facilitators to smartwatch use, motivational texts, instructional educational modules, engaging in video-guided physical rehabilitation, walking exercise, and co-participating with a care partner. While we estimate conducting 10 to 20 interviews each for those with and without mild AD/ADRD, the exact number will be guided by the achievement of thematic saturation [68,69].

We will use purposive sampling with maximum variation [70,71] by AD/ADRD status (mild AD/ADRD vs. no AD/ADRD) to ensure diverse perspectives. We will use a structured interview guide to guide interviews. Interviews will be audio-recorded and transcribed verbatim using a secure, institutionally approved automated transcription platform. All transcripts will be reviewed against the original audio files by trained study personnel to ensure accuracy and to correct transcription errors, particularly for medical terminology or participant-specific phrasing. During this review, transcripts will be systematically de-identified by removing or replacing names, locations, and other potentially identifying information. Audio recordings and de-identified transcripts will be stored on secure, access-restricted institutional servers, and only approved study personnel will have access to study data.

De-identified transcripts will be analyzed using a constructivist grounded theory approach [72,73,74], including pre-coding, in vivo coding, focused coding, constant comparative analysis, iterative codebook revision, and assessment of saturation [69,75]. Dual coding by the principal investigator and a skilled research assistant, along with audit trails and reflective memoing, will ensure the credibility, dependability, confirmability, member checking, and transferability of the findings [76,77]. The analysis will generate a conceptual model of effective implementation of ACTIVE dyadic intervention, which will be used to refine the intervention and inform the design of a future definitive trial.

### 2.14. Ethical and Safety Considerations

This study was approved by the NYU Langone Health Institutional Review Board (IRB# i25-01158; 22 December 2025) and is registered on ClinicalTrials.gov (NCT07321587; Registration Date: 23 December 2025) [78]. All participants will provide electronic informed consent prior to participation. We will conduct the study in accordance with all applicable ethical guidelines for human subject research and comply with institutional and federal guidelines. To protect participant safety and confidentiality, all research personnel have completed comprehensive training in human subject protection. All data will be stored securely, de-identified when appropriate, and accessed only by authorized study staff. Any substantive protocol amendments will be reviewed and approved by the IRB, updated on ClinicalTrials.gov as applicable, and communicated to study participants when relevant. This protocol follows the Standard Protocol Items: Recommendations for Interventional Trials (SPIRIT) guidelines [79].

Given the minimal-risk, behavioral nature of the ACTIVE intervention, no formal Data Monitoring Committee is required. Interim analyses will be conducted intermittently for participant monitoring. Trial conduct, data quality, and participant safety will be monitored on an ongoing basis by the study principal investigator and designated study staff in accordance with IRB-approved procedures. Any unanticipated problems or adverse events will be reported promptly to the IRB in accordance with institutional policies.

The study team will continuously assess the potential benefits and harm associated with the study. The potential benefits include improved physical activity engagement, reduced fall risk, and enhanced quality of life among older adult–care partner dyads with and without mild AD/ADRD. Potential harms, including physical, psychological, and technical adverse events, will be systematically monitored throughout the study. Feasibility outcomes will help identify unintended challenges or barriers, ensuring a careful assessment of the intervention’s positive and negative impacts.

## 3. Discussion

This pilot study is designed to evaluate the feasibility and preliminary efficacy of the ACTIVE intervention, a novel, theory-guided, multi-component, dyadic digital health intervention targeting physical activity engagement, fall risk, and quality of life among community-dwelling older adults with and without mild AD/ADRD and their care partners. The study addresses a critical gap in fall prevention and physical activity promotion research by integrating wearable technology, video-guided physical rehabilitation, educational content, motivational messaging, and structured care partner involvement into a single, scalable intervention delivered entirely in the home setting.

The expected findings will contribute to the growing body of evidence supporting digitally delivered, community-based physical activity interventions for older adults, particularly those at elevated risk for falls and functional decline [80,81,82,83]. Feasibility outcomes will be particularly informative for future trial design. Demonstrating adequate recruitment, retention, and adherence among dyads, including those with mild AD/ADRD, would provide strong justification for inclusion of this high-risk population in larger digital health trials. Similarly, detailed engagement metrics captured through myACTIVEsteps.com and Fitabase will allow identification of which intervention components are most strongly associated with sustained participation, informing optimization and potential streamlining of the intervention for broader dissemination. Although this pilot is not powered to detect definitive clinical effects, preliminary efficacy estimates will provide critical signals regarding the potential impact of ACTIVE on objective physical activity metrics, fall-related outcomes, functional independence, and quality of life. Improvements in physical activity behaviors, even over a short intervention period, may support the plausibility of longer-term benefits, aligning with prior studies demonstrating sustained effects of physical activity and digital health interventions among older adults with longer follow-up [81,84,85]. The qualitative component of this study will further contextualize quantitative findings by elucidating barriers and facilitators to engagement with each intervention component. Insights from care recipients and care partners will inform refinements related to messaging frequency, technological burden, cognitive accessibility, and the balance between independence and supervision. The result of this pilot study will directly inform the design of a future definitive effectiveness–implementation hybrid trial.

This pilot study has its limitations. The small sample size and short intervention period limit our ability to draw definitive conclusions about efficacy or long-term outcomes, but are appropriate for establishing feasibility, acceptability, and preliminary effect sizes to inform sample size calculations for a larger trial. Recruitment through ResearchMatch may enrich the sample with individuals who are more technologically comfortable or health-motivated, reducing generalizability to the broader population of community-dwelling older adults. Older adults with mild AD/ADRD may face unique cognitive and attentional challenges that could affect comprehension of instructions, consistent device use, and accuracy of self-reported outcomes. At the same time, care partners’ engagement may vary substantially based on competing responsibilities and caregiver burden. While the requirement for a co-residing or nearby care partner was included to ensure adequate exposure to intervention and safety monitoring, it may limit generalizability. The study relies on self-reported survey measures, which may introduce recall and social desirability bias, as well as discrepancies between perceived and actual behavior [86,87]. To mitigate recall and social desirability bias among participants with mild AD/ADRD, we will employ several strategies. Care partners will be permitted to assist with recall when needed, though we acknowledge this may introduce proxy reporting considerations. To address social desirability bias, we will emphasize the confidentiality of responses and use neutral, non-judgmental language during data collection. Where possible, self-reported measures will be triangulated with objective data captured on Fitabase (e.g., physical activity levels) and myACTIVEsteps.com (e.g., program engagement metrics) to cross-validate responses and reduce reliance solely on self-report.

The crossover design, while improving internal validity and allowing within-participant comparisons with fewer total participants, may introduce learning or carryover effects that complicate the interpretation of intervention-related changes. While the requirement for a co-residing or nearby care partner was included to ensure adequate exposure to intervention and safety monitoring, it may limit generalizability. In addition, dyadic-level interdependence may influence outcomes in ways that cannot be fully isolated with the pilot sample size. Finally, forgetting to wear the smartwatch every day and to charge it twice a week may introduce missing data, which may be difficult to impute depending on the pattern and volume of missingness. Despite these limitations, this pilot study lays a foundation for a scalable intervention that may improve engagement in physical activity and the quality of life of older adults and their care partners, including those with mild AD/ADRD.

The ACTIVE intervention has the potential to enhance physical activity, functional independence, and overall well-being among older adults and their care partners. At the conclusion of the ACTIVE pilot study, we plan to share the study findings with participants, care partners, and relevant stakeholders through plain-language summaries. Participants who express interest will receive a concise summary of results via email or mail. In addition, study outcomes will be posted on the ClinicalTrials.gov registry in accordance with reporting requirements. Findings will also be disseminated through peer-reviewed publications and presentations at scientific meetings to inform the broader research and clinical community.

## 4. Conclusions

This pilot study will provide foundational evidence on the feasibility and preliminary efficacy of the ACTIVE intervention in older adults and their care partners. Findings will guide optimization of the intervention, inform the design of a larger trial, and ultimately support the development of strategies to promote safe, sustained physical activity and functional engagement among older adults.

## Figures and Tables

**Table 1:** Components of the ACTIVE Intervention.

Activity Tracking, Care Partner Co-Participation, Text Reminders, Instructional Education, Video-Guided Physical Rehabilitation, and Exercise	
Component	What It Includes
Activity Tracking	Fitbit Inspire 3 wearable, continuous step count and activity intensity monitoring, synced weekly
Care Partner Co-Participation	Care partner joining to supervise for safety and engage in home exercise, provide encouragement, and assist with technology
Text Reminders	Automated daily motivational text messages tailored to preceding day’s activity
Instructional Education	Digital education modules (short videos + PDFs): safe exercise, fall prevention
Video-Guided Physical Rehabilitation	Scheduled remote PT via video platform: Week 1: three sessions (gait, balance, strength) Weeks 2–4: weekly check-ins with progression Tailored exercise plans provided electronically
Exercise	Daily walking goal (≥15 min), tracked with wearable; progressive increase based on baseline activity

**Table 2:** Summary of the weekly schedule of the ACTIVE Intervention.

Time Point	Dyad Roles
Weeks 1: Pre-intervention	Control + Intervention Dyad: Mailing of printed educational infographics and smartwatch Troubleshooting to ensure data capture
Weeks 2–4	Control + Intervention Dyad: 1. Weekly dyad telephone check-in by research staff 2. Continuous tracking of activity Intervention Dyad Alone: 3. Two-weekly 3 min educational videos 4. Three-weekly video-guided physical rehabilitation schedule 5. Daily motivational texts
Week 5: Cross-over	Switch roles Interviews and Surveys Troubleshooting to ensure data capture
Weeks 6–8	Control + Intervention Dyad: 1. Weekly dyad telephone check-in by research staff 2. Continuous tracking of activity Control Dyad Alone: 3. Two-weekly 3 min educational videos 4. Three-weekly video-guided physical rehabilitation schedule 5. Daily motivational texts
Week 9–10	Interviews and Surveys

ACTIVE: Activity tracking, Care partner co-participation, Text reminders, Instructional education, Video-Guided Physical Rehabilitation, and Exercise—walking.

**Table 3:** Summary of outcome and covariate variables, including sources of data and how each measure is defined.

Outcome Measures	Analysis	Data Source
Recruitment		
1. % of dyad recruited 2. % of dyad randomized	Benchmark: ≥75%	REDCap REDCap
Adoption (measured in week 1)		
1. Smartwatch use ≥ 3 days	Benchmark: ≥75%	Fitabase
2. Motivational Texts ≥ 1 opened		Fitabase
3. Virtual PT videos ≥ 1 sessions		myACTIVEsteps
Adherence (measured weeks 1–3)		
1. Smartwatch use: % days ≥ 8 h	Benchmark: ≥75%	Fitabase
2. Videos: % sessions completed		myACTIVEsteps
3. Virtual PT: % completed		myACTIVEsteps
Acceptability (Week 5 and 9)		
1. Website SUS score 2. Virtual PT SUS Score	Benchmark: Mean ≥ 68	REDCap REDCap
1. Smartwatch TAM Score	Benchmark: Mean ≥ 4.0	REDCap
2. Motivational Texts TAM Score		REDCap
3. Educational Videos TAM Score		REDCap
4. Virtual PT TAM Score		REDCap
Fidelity (Week 5 and 9)		
1. % motivational text sent 2. % educational text notification sent	Benchmark: ≥90%	Fitabase Fitabase
Retention		
1. % of dyad completed the study	Benchmark: ≥75%	REDCap
Preliminary Effectiveness (Week 5 and 9)		
1. Activity: MET-mins/week	Linear Mixed Effect Model	Fitabase
2. FES-I score pre/post		REDCap
3. ADL/IADL score		REDCap
4. SF-12 pre/post		REDCap
5. KAP Scores–pre/post		REDCap
Covariate Measures (Assessed at Baseline)		
Sociodemographic: Age, Sex, Race/Ethnicity, Health Insurance, Education, Marital Status, Living Situation		REDCap
Health Measures: Clinical Frailty Scale, Charlson Comorbidity Index,		REDCap

PT: Physical Therapy; SUS: System Usability Scale; TAM: Technology Acceptance Model; MET: Metabolic Equivalent of Task; FES-I: Fall Efficacy Scale-International; ADL/IADL: Activities of Daily Living/Instrumental Activities of Daily Living; SF: Short Form; REDCap: Research Electronic Data Capture.

## Data Availability

No new data were created or analyzed in this study.

## Abbreviations

The following abbreviations are used in this manuscript:

ACTIVE	Activity Tracking, Care Partner Co-Participation, Text Reminders, Instructional Education, Video-Guided Physical Rehabilitation, and Exercise
AD/ADRD	Alzheimer’s disease and Alzheimer’s disease-related dementias
STEADI	Stopping Elderly Accidents, Deaths, and Injuries
SCT	Social Cognitive Theory
REDCap	Research Electronic Data Capture
